# Do railway lines affect the distribution of woodland birds during autumn?

**DOI:** 10.1371/journal.pone.0231301

**Published:** 2020-04-15

**Authors:** Jarosław Wiącek, Marcin Polak, Maciej Filipiuk, Marek Kucharczyk, Łukasz Dawidowicz

**Affiliations:** 1 Department of Zoology and Nature Conservation, Institute of Biological Sciences, Maria Curie-Skłodowska University, Lublin, Poland; 2 Droga Męczenników Majdanka, Lublin, Poland; Universidad Miguel Hernandez de Elche, SPAIN

## Abstract

Research results on the impact of railway noise on birds show a variety of bird responses. These behaviours are often different from those exhibited by birds occupying habitats along tarred roads. Knowledge of this subject is still incomplete. We attempted to define the influence of a heavily transited railway line on bird communities at stopover sites near the tracks during the autumn migration period. Birds were counted using the point method at 45 observation points located at three distances (30 m, 280 m, 530 m) from the tracks. At each point we determined the habitat parameters and the intensity of noise. A total of 614 individuals from 29 species were recorded on the study plot. The results of our observations indicate that the railway line does not adversely affect woodland birds during the autumn migration. The results showed that the abundance of birds and the species richness were actually the highest near the railway line. Species foraging on invertebrates preferred the neighbourhood of the tracks. The number of the most common species did not differ widely in relation to distance from the tracks. These data may be helpful in planning and managing the environment in the context of bird conservation, protection from railway noise and collisions with trains.

## Introduction

The world economy is based on the movement of raw materials, products and people, and railways play an important role in the global transport network. Promoted by different countries for their economic and environmental advantages over other means of transport [1], railways are playing an increasing part in both developed and developing countries [2]. However, along with expanding urbanization, railway networks are adversely affecting the natural environment [3]. Animals living in the neighbourhood of railway lines are disturbed by noise and light, and are susceptible to collisions with passing trains, electric catenaries and other elements of railway infrastructure [1]. Recent studies have shown that noise pollution in woodland habitats near roads and railway lines is an important problem [4,5]. Noise from road traffic significantly decreases the numbers and species diversity of birds in the vicinity of roads frequented by large numbers of motor vehicles [6,7]. Railway noise can affect birds in other ways, however, depending on the habitat. In the Netherlands, a significant reduction in bird density was found in meadows close by railway lines [8]. But the proximity of railway lines can have a much more serious impact. For example, birds flying slowly without performing rapid flight manoeuvres or young inexperienced individuals often die as a result of colliding with rail infrastructure or passing trains [3,9,10]. Migrating birds are the most seriously endangered by such collisions, especially during autumn. Young birds flying during the night, nomadic species or birds that move in flocks most often lose their lives in such circumstances [11].

In contrast to the declines in meadow birds near railway lines in the Netherlands [8], our earlier study carried out in eastern Poland showed that the species richness of woodland birds was higher close to railway tracks [12]. Environmental factors like the much more complex habitat structure, the greater availability of nest niches and the richer food resources at the edge of woodland encourage birds to start breeding there. Ecotone habitats in transport corridors are much more mosaic-like than homogeneous habitats, so they are quickly colonized by different species of animals, including birds [12,13,14,15,16]. Several studies have documented biotic and structural homogenization in such edge habitats, but the high levels of insolation and rich habitat structures along borders between woodlands and transport corridors provide perfect conditions for invertebrates (especially insects), insectivorous mammals, rodents [17, 18, 19] and birds foraging on these groups of animals [11,16]. Some species of birds, especially generalists, may benefit from the “edge effect”, but the species diversity of forest specialists may be lower along woodland edges [20].

Most investigators have focused on the impact of railway lines on birds during the breeding season; in contrast, the present research addresses the impact of railway lines on birds during autumn. The autumn migration, taking up about one third of the year, plays a very important role in the annual life cycle of birds [21]. On migration, birds spend long periods at stopover sites, feeding and resting up to 95% of the time [22,23,24]. Moreover, they are exposed to many dangers: they compete for food, and try to avoid predators and other term related with “human disturbance” [25,26,27]. Human activities in general has a negative effect on animals, positive examples can be found. For example, some research has indicated that despite anthropogenic pressure, many animals, including birds, live in the neighbourhood of humans and their infrastructure [16,28,29,30]. The areas around railway lines and their infrastructure offer favourable conditions for birds during the breeding season [5,16,30] migration (this study) and wintering [31]. The high diversity of habitats available in transport corridors is a particularly important factor encouraging birds to nest near railway lines. In particular, the edge effect along the border between a transport corridor and woodland creates a very attractive habitat for some species of birds, which achieve a greater density and species richness in these places, although the success of these species does depend on their specific ecological requirements [12]. In contrast, many species may be negatively affected by the edge effect [5,20,32]. Birds from different ecological guilds occupying areas adjacent to railway lines may react differently to passing trains and the noise they produce [12]. The diverse ecological niches situated along road/railway—woodland borders may be colonized by birds even though the risk of collisions with vehicles/trains is high. These habitats are attractive because they offer abundant food resources, roosting sites, and a lower probability of attacks by predators [16,32].

The aim of our research was to investigate whether a railway line affects the number of individual birds and the number of bird species during autumn. The null hypothesis assumed that the influence of the presence of a high traffic railway on the distribution of birds in the study area would be neutral. We expected that birds would prefer the vicinity of the line, as had been the case during the breeding season [5]. Therefore the alternative hypothesis assumed that, in view of the positive effects of the railway that we noted in previous studies, we should expect increasing numbers and species richness of birds in its vicinity[5,31].

## Methods

Our study was carried out in the field. The tests were not invasive to birds and other animals. No special permission was required. Government forests in Poland are open for scientist and scientific studies.

### 1. Study area

The study plot was located in the Puławy Forest District (N 51° 50ˊ 02˝, E 21° 91ˊ 94˝) in eastern Poland. The main habitat in the plot was pine forest with predominant Scots pine (*Pinus sylvestris*) and admixtures of silver birch (*Betula pendula*). The field work was carried out along the Warsaw-Lublin railway line between the towns of Puławy and Deblin. The intensity of traffic along this line during the study was 144 trains per 24 h (111 passenger and 33 freight trains). The average speed of passenger trains was about 70km/h, while the speed of freight trains was about 40 km/h. The line runs through this woodland in a 50 m wide corridor. The study area was situated on the north-eastern side of the tracks.

### 2. Bird surveys

**Fig 1:**
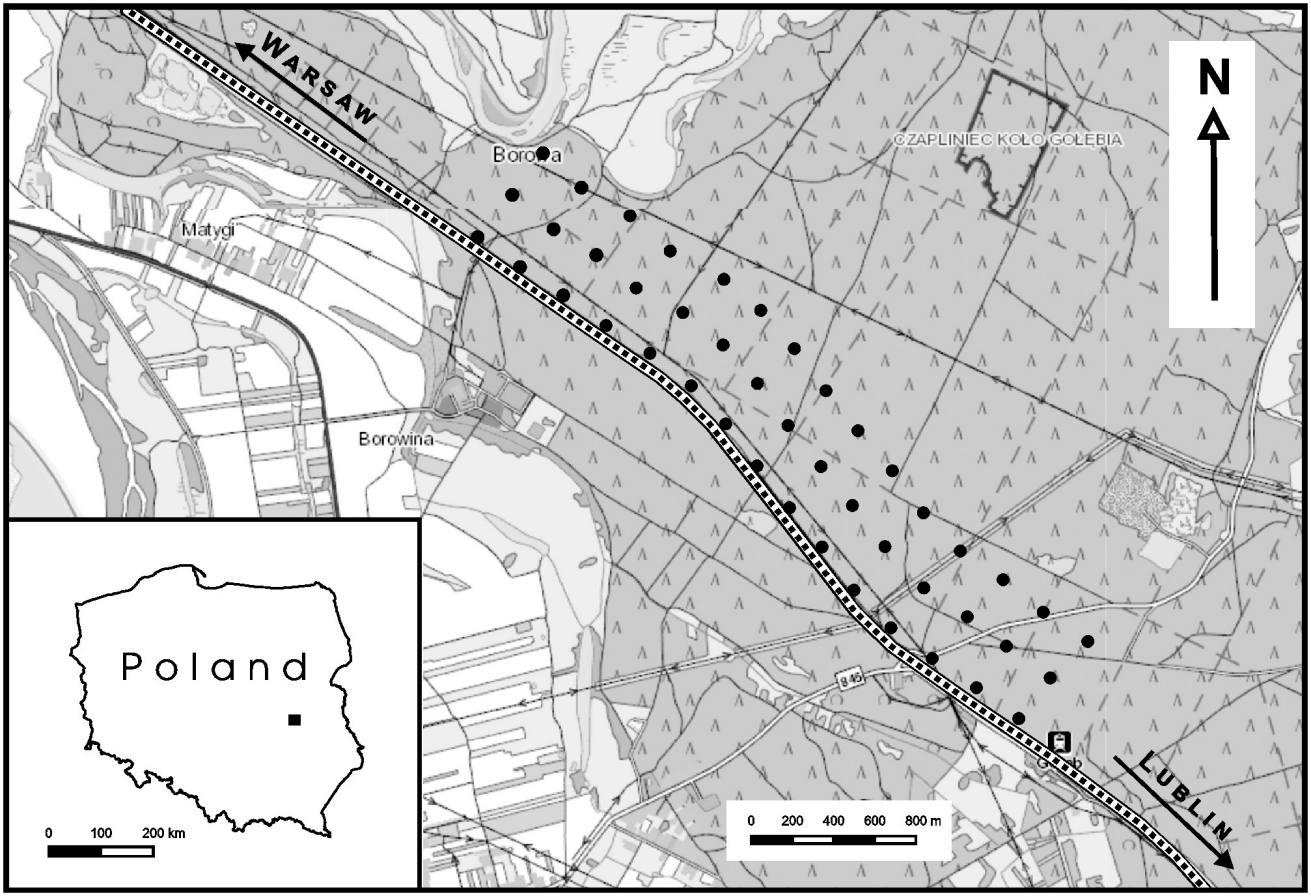
Map of the study plot showing the point-count locations (45 black dots) near a railway line in the Puławy Forest in eastern Poland.

The numbers and diversity of woodland birds were determined using the point-count method [33] at 45 observation/listening points located along three rows running parallel to the railway (Fig 1). All the points were established and recorded on GPS receivers in April before the start of counting. The first row of 15 points (henceforth referred to as points A) lay 30 m from the railway, the next row of 15 points (points B) was situated 280 m from the railway, and the last row of 15 points (points C) ran at a distance of 530 m from the railway. All the points were 250 m apart from one another. Counting at each point lasted for 5 minutes. All the birds seen and heard within a radius of 100 m were recorded, but birds flying over the study plot were disregarded. Three counts were done at each point—on 30 September 2015, 19 October 2015 and 25 November 2015—all between 06:30 and 09:00 hrs (local time). The limiting factor in this type of study is the fact that the observers themselves sometimes have difficulty in hearing the birds above the noise of the trains, so some will undoubtedly have gone unrecorded [34,35]. Nevertheless, we were aware of these limitations during our point counts and tried to minimize them. Our task was made a little easier because the traffic along the railway was not continuous, and we were able to record the birds’ vocal activity in the gaps between passing trains. The division into feeding guilds was based on previous published papers [5,32,36,37]. Owing to the small sample size, raptors were not included in the ecological guild analyses. The counts at all the points on one day were performed by three of four experienced observers (JW, MF, ŁD and MP), who walked parallel to one other, each along a different row of points (A, B, C). In order to minimize the observer effect, during each successive count, the observers walked along a different row of points. The measure of species richness in relation to the distance from the railway was taken to be the sum of all species come across during the three counts, and the number of birds was the sum total of all individuals discovered during all three counts. In other cases each survey date was treated as an independent sample for comparison.

### 3. Environmental variables

Before the counts were started, the study plot was selected very carefully in order to reduce to an absolute minimum the effect of environmental parameters on bird clustering. The plot was located in the depths of a large, dense forest complex (Fig 1). The noise of trains and the vegetation structure at every listening point was assessed with the aid of environmental parameters (see Table 1).

**Table 1:** Habitat variables obtained at the observation/listening points.

Variable	Meaning
Canopy cover (%)	% canopy cover in eleven categories: 0–0%; 10–1–10%; 20–11–20%; 30–21–30%; 40–31–40%; 50–51–60%, 60–61–70%, 70–71–80%, 80–81–90%, 90–91–99%, 100–100% within a circle of 30 m radius
Tree height (m)	Mean height (measured with an altimeter) of 5 trees growing within a circle of 30 m radius
Number of deciduous trees	The number of deciduous trees among the nearest 30 trees with a diameter at breast height >20 cm
Number of dead trees	The number of dead trees with a diameter at breast height >20 cm within a circle of 50 m radius
Density of shrubs	Number of shrubs growing within a circle of 30 m radius
Shrub cover (%)	% shrub cover in eleven categories: 0–0%; 10–1–10%; 20–11–20%; 30–21–30%; 40–31–40%; 50–51–60%, 60–61–70%, 70–71–80%, 80–81–90%, 90–91–99%, 100–100% within a circle of 30 m radius
Herb cover (%)	% herb cover in eleven categories: 0–0%; 10–1–10%; 20–11–20%; 30–21–30%; 40–31–40%; 50–51–60%, 60–61–70%, 70–71–80%, 80–81–90%, 90–91–99%, 100–100% within a circle of 30 m radius
Herb height (cm)	Mean height of the herbaceous vegetation at 5 sites selected at random within a circle of 30 m radius

Direct measurements of sound levels during the counts were applied to determine the model of noise propagation across our study area. The level of railway noise was measured at all the points during every count in September, October and November using a CHY 650 digital sound level meter (IEC 651–1979 Type 2, ANSI S1.4–1983 Type 2, JIS C 1502). The noise level at the centre of each point was measured for 5 minutes and the highest level recorded. Railway noise was measured on weekdays in comparable and stable weather (no rain or high winds).

### 4. Data analyses

Parametric tests were applied to the analyses after the Kolmogorov-Smirnov test (*P* > 0.05) had been used to check whether the distribution was consistent with the normal. A bilateral critical region was assumed in the tests, and the results were deemed significant if the probability of committing an error of the first kind was ≤ 0.05.

Analysis of variance (ANOVA) was used to assess the propagation of noise over the study area and to determine the differences in species richness and numbers along rows of observation points at three different distances from the tracks (30 m, 280 m, 530 m).

Redundancy analysis (RDA) was used to analyse the relationship between the numbers of particular bird species and distance from the railway line, the latter factor being the environmental predictor in this analysis. The test was carried out to detect the species’ preferences for being near the tracks or in the depths of the forest. The Monte Carlo test with 500 permutations was used to determine the significance of the canonical axes. Only species with abundances ≥ 10 individuals were included in the RDA (see Table 2).

**Table 2:** Forest bird community composition in relation to distance from the railway line in eastern Poland. Bird species were classified according to their foraging (G—granivore-insectivore; I—insectivore; R—raptor) and migratory behaviour (M-migratory; R-resident species). For each species, we show the total number of birds detected and the percentage (in parentheses) at each point (A—30 m, B—280 m, C—530 m).

Species	Foraging guild	Migratory guild	Total number	Number of individuals (%)		
				points A (*n* = 15)	points B (*n* = 15)	points C (*n* = 15)
*Parus major*	I	M	157 (25,6)	65 (41)	68 (43)	24 (16)
*Regulus regulus*	I	M	67 (10,9)	31 (46)	20 (30)	16 (24)
*Dendrocopos major*	G	R	52 (8,5)	11(21)	19 (37)	22 (43)
*Spinus spinus*	G	M	50 (8,1)	14 (28)	22 (44)	14(28)
*Turdus viscivorus*	G	M	40 (6,5)	6 (15)	30 (75)	4 (10)
*Garrulus glandarius*	G	M	37 (6)	24 (65)	11 (28)	2 (7)
*Lophophanes cristatus*	I	R	35 (5,7)	14 (40)	5 (14)	16 (46)
*Fringilla coelebs*	G	M	24 (3,9)	9 (38)	15 (62)	0 (0)
*Cyanistes caeruleus*	I	M	19 (3,1)	7 (37)	8 (42)	4 (21)
*Pyrrhula pyrrhula*	G	M	19 (3,1)	0 (0)	2 (11)	17 (89)
*Periparus ater*	I	R	18 (2,9)	7 (39)	4 (22)	7 (39)
*Poecile montana*	I	R	15 (2,4)	10 (67)	1 (7)	4 (27)
*Troglodytes troglodytes*	I	M	14 (2,3)	6 (43)	7 (50)	1 (7)
*Corvus corax*	G	M	13 (2,1)	6 (46)	0 (0)	7 (54)
*Erithacus rubecula*	I	M	9 (1,5)	8 (89)	1 (11)	0 (0)
*Chloris chloris*	G	R	9 (1,5)	0 (0)	0 (0)	9 (100)
*Turdus pilaris*	I	M	6 (0,9)	1 (17)	5 (83)	0 (0)
*Turdus merula*	I	M	6 (0,9)	3 (50)	3 (50)	0 (0)
*Picus viridis*	I	R	4 (0,6)	0 (0)	1 (25)	3 (75)
*Dryocopus martius*	I	R	4 (0,6)	3 (75)	0 (0)	1 (25)
*Turdus philomelos*	I	M	3 (0,5)	3 (100)	0 (0)	0 (0)
*C*. *coccothraustes*	G	M	3 (0,5)	2 (67)	1 (33)	0 (0)
*Certhia familiaris*	I	R	2 (0,3)	1 (50)	0 (0)	1 (50)
*Lullula arborea*	I	M	2 (0,3)	2(100)	0 (0)	0 (0)
*Buteo buteo*	R	M	2 (0,3)	1 (50)	0 (0)	1(50)
*Accipiter nisus*	R	M	1 (0,2)	0 (0)	1 (100)	0 (0)
*Phylloscopus trochilus*	I	M	1 (0,2)	1 (100)	0 (0)	0 (0)
*Phylloscopus collybita*	I	M	1 (0,2)	1 (100)	0 (0)	0 (0)
*Carduelis carduelis*	G	R	1 (0,2)	0 (0)	0 (0)	1 (100)
Total			614	236	224	154

Multiple regression analysis was conducted to test the impact of eight environmental parameters (canopy cover, tree height, number of deciduous trees, number of dead trees, shrub cover, density of shrubs, herb cover and herb height) on the abundance and number of species (see suplementary material 1). Therefore, multivariate analysis of variance (MANOVA) was performed to test for variation in avian feeding groups (insectivorous and granivorous guilds) at points located at different distances from the railway.

A Generalized Linear Model (GLZ with log-link function and Poisson error distribution) was used to estimate the influence of the distance from the railway line on bird abundance and avian species richness in the vicinity of the railway line.

The computations (Kolmogorov-Smirnov test, ANOVA, MANOVA, GLZ and multiple regression) were performed using STATISTICA 12.0 [38]; RDA was carried out using Canoco 4.0 [39].

## Results

### Environmental parameters

The mean noise intensity during the counts was 52.6 ± 18.22 dB (range 33.5–101.1 dB; *n* = 45) in September, 54.1 ± 18.68 dB (33.6–96.1 dB; *n* = 45) in October and 58.9 ± 17.7 dB (35.5–98.8; *n* = 45) in November. Significant differences were demonstrated in noise propagation between the point categories during the counts in September (ANOVA; *F*_2, 42_ = 9.89; *P* < 0.001), October (*F*_2, 42_ = 9.82; *P* < 0.001) and November (*F*_2, 42_ = 7.20; *P* < 0.005), (suplementary material 2). Analysis of the environmental parameters showed that three of the eight habitat parameters differentiated the study area (suplementary material 1). The number of deciduous trees and herb cover decreased with distance from the railway, and only canopy cover increased with distance from the railway.

### Numbers of individuals

**Fig 2:**
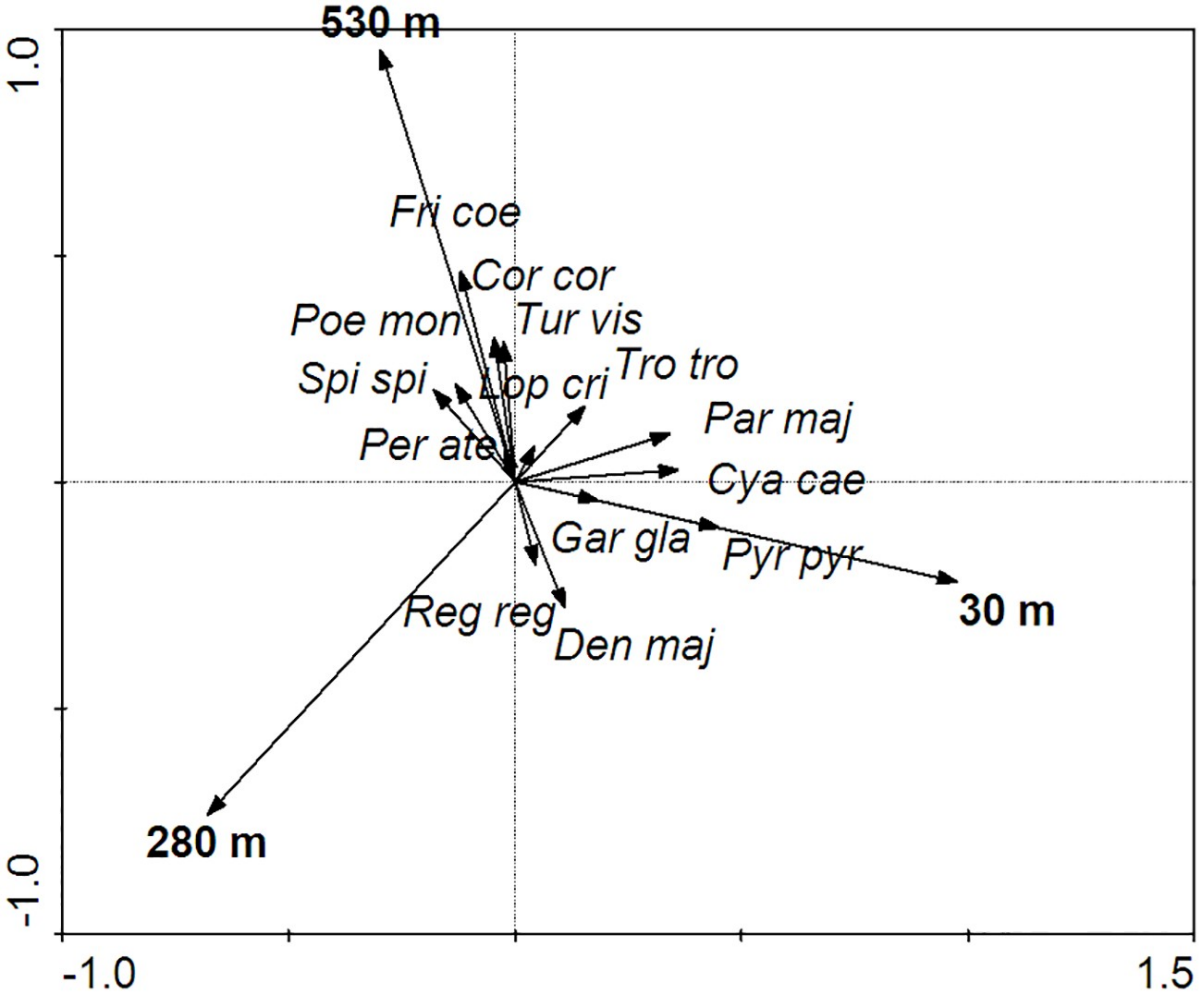
Ordination diagram of RDA analysis with the most common bird species in relation to distance from the railway line. The species name abbreviations consist of the first letters of the generic and specific names.

In all, 614 birds from 29 species (Table 2) were recorded during the three counts. The most numerous species was great tit (*Parus major)*, which made up 25.6% of the birds counted in the study area. The dominants (≥ 5%) also included goldcrest (*Regulus regulus)*, great spotted woodpecker (*Dendrocopos major)*, siskin (*Spinus spinus*), mistle thrush (*Turdus viscivorus*), Eurasian jay (*Garrulus glandarius*) and crested tit (*Lophophanes cristatus*). The numbers of the most common birds (>10 inds.) did not differ widely in relation to distance from the railway (Fig 2: Monte Carlo test of the significance of the first axis; *F* ratio = 2.84; *P* = 0.15; Monte Carlo test of the significance of all axes; *F* ratio = 1.63; *P* = 0.11). Multiple regression analysis indicated that canopy cover (*B* = -0.438; *SE* = 1.04; *P <* 0.05) was the only one of the eight environmental parameters (in all cases *P* = 0.15) influencing abundance (all individuals/point).

**Fig 3:**
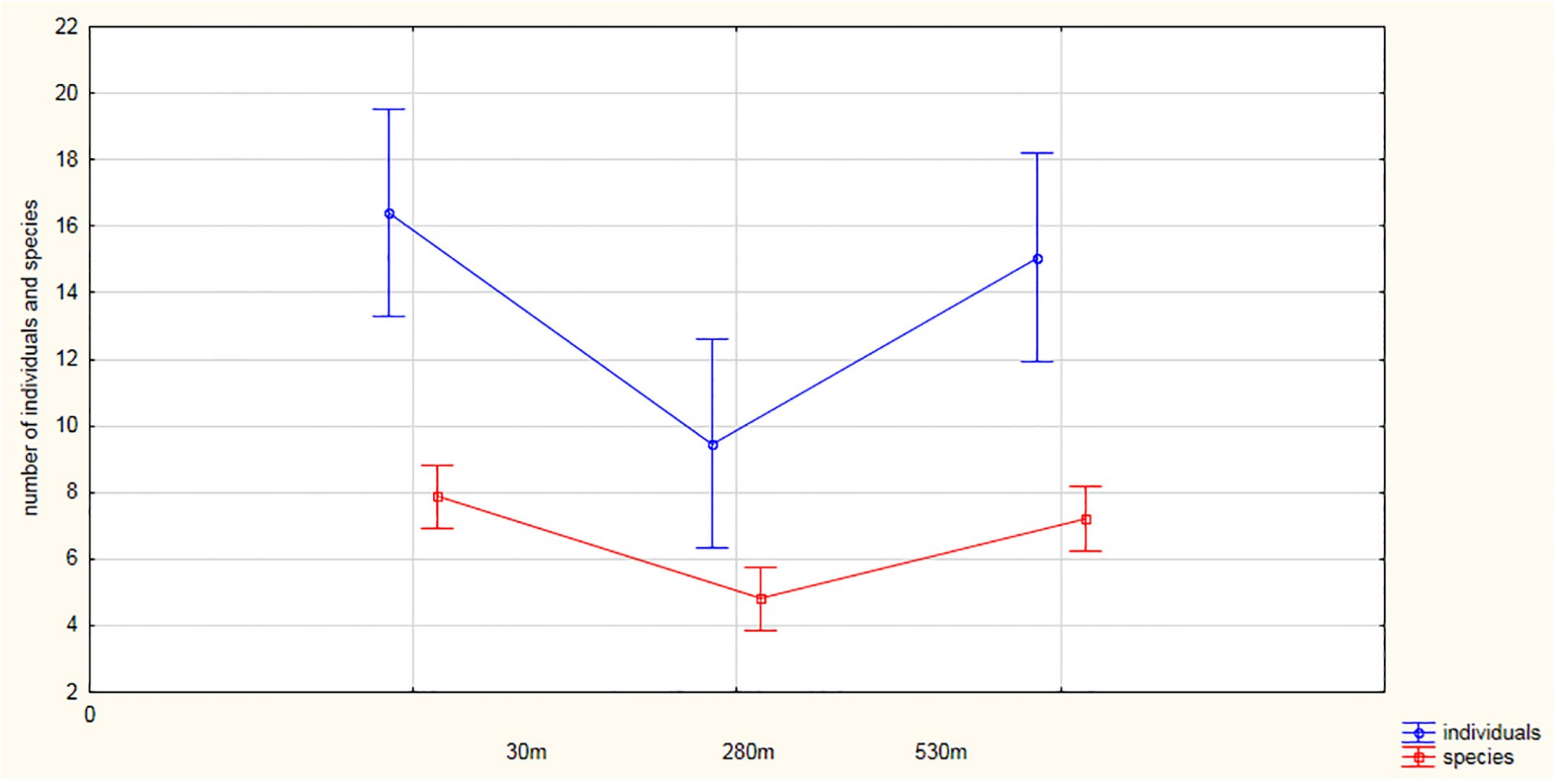
Relationship between the number and birds species richness at the point-count locations in relation to distance from the railway line.

The differences between the mean bird abundances (all individuals/point) in the three rows in September were not significant (5.28 ± 3.88 (range 1–19; *n* = 45; ANOVA: *F*_2, 42_ = 0.68, *P* = 0.51), but they were significant in October (4.95 ± 4.53 (range 0–18; *n* = 45; ANOVA *F*_2, 42_ = 4.39, *P* < 0.05) and November (3.55 ± 3.36 (range 0–15; *n* = 45; ANOVA; *F*_2, 42_ = 4.54; *P* < 0.05)). The mean number of birds at points A was 16.4 ± 5.4 (range 9–26; *n* = 15) and was higher than the numbers at points B (9.46 ± 4.15; 3–19; *n* = 15) and C (15.1 ± 7.7; 5–29; *n* = 15), (Fig 3). There were significant differences between the numbers of individuals in the various rows (Generalized Linear Model; Estimate = - 0.34; Wald statistic = 28.06; *P <* 0.001).

### Numbers of species

The number of species per point was the highest near the railway (ANOVA; *F*_2, 42_ = 11.339; *P <* 0.001). The mean number of species at points A, lying closest to the railway, was 7.86 ± 1.59 (range 5–10, *n* = 15) and differed significantly from the number at points B (4.8 ± 1.93; range 2–9; *n* = 15). There were 7.2 ± 2.01 species (range 3–10; *n* = 15) in the last row of points (C), (Fig 3). There were significant differences between the numbers of species in the different rows (Generalized Linear Model; Estimate = - 0.30; Wald statistic = 11.039; *P* = 0.001) and there were also significant differences between the mean number of species in September (3.17 ± 1.54 (range 1–6; *n* = 45; ANOVA; *F*_2, 42_ = 4.15; *P* < 0.05), October (2.57 ± 1.34 (range 0–6; *n* = 45; ANOVA; *F*_2,42_ = 3.39; *P* < 0.05) and November (2.26 ± 1.56 (range 0–6; *n* = 45; ANOVA; *F*_2, 42_ = 6.48; *P* < 0.01). Canopy cover (*B* = - 0.41; *SE* = 0.358; *P <* 0.05) was the only environmental factor (multiple regression; in all cases *P* > 0.15) influencing species diversity.

### Ecological guilds

**Fig 4:**
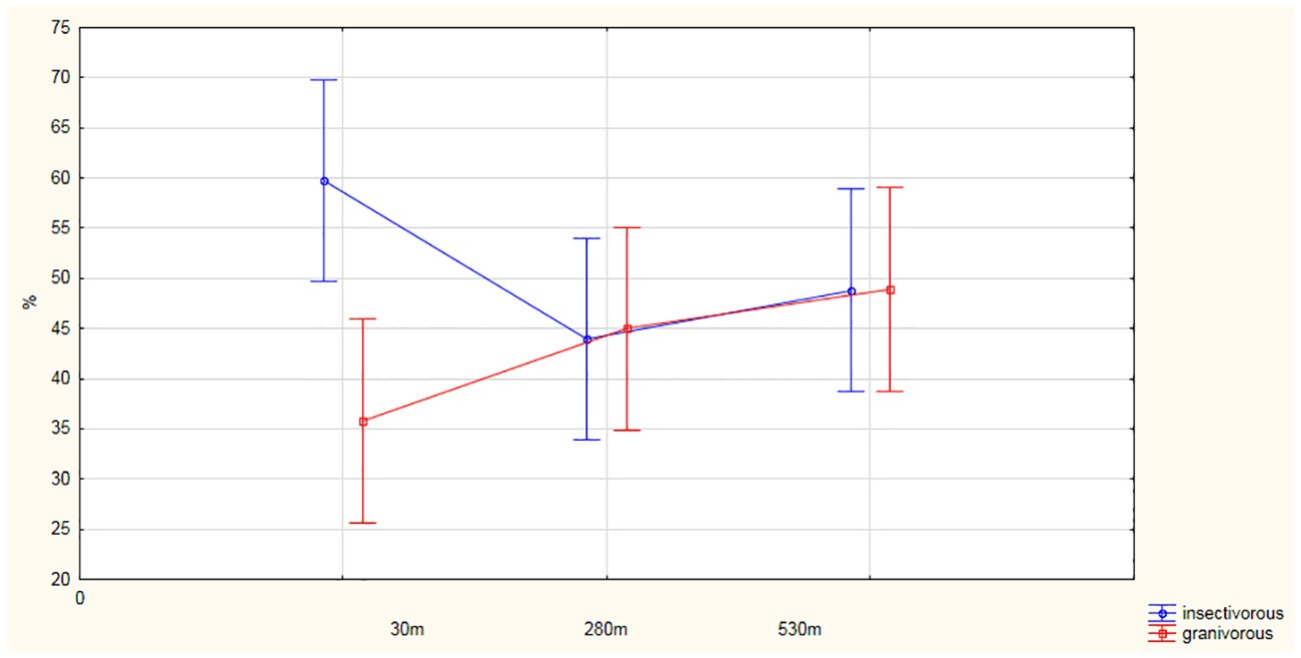
Foraging (Insectivorous; Granivorous-insectivorous) guilds in relation to distance from the railway line.

**Fig 5:**
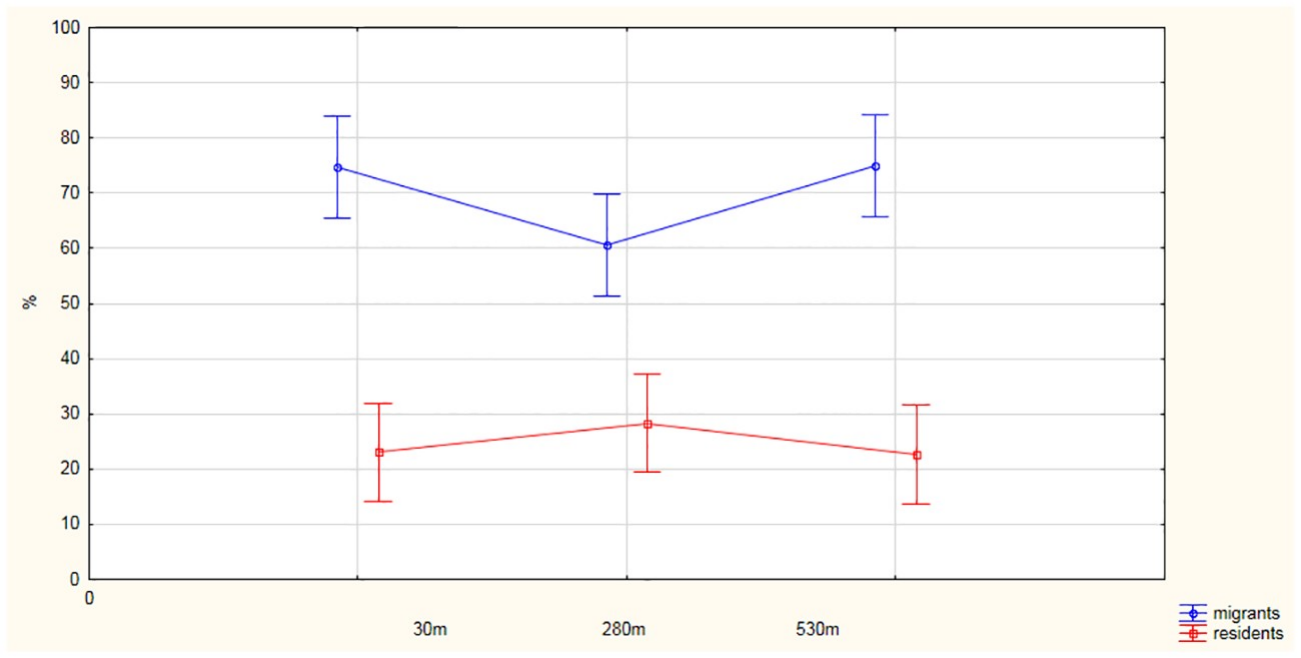
Migratory and resident guilds in relation to distance from the railway line.

More insectivorous species were observed close to the railway line, whereas the numbers of granivores increased with distance from the tracks, but these differences between rows A, B and C were not significant (Fig 4, MANOVA F_4, 25_ = 1.97; P = 0.09). The numbers of individuals foraging on invertebrates decreased during the successive months of the study (September, October and November), but the numbers of granivores increased; these data were statistically significant (MANOVA F_(4,250)_ = 2.7; P = 0.03), (suplementary material 3). Similarly, the number of migrant species from month to month, whereas resident species increased significantly (MANOVA F_4, 25_ = 5.99; P = 0.0001),(suplementary material 4). There was no statistical relationship in the distribution of the two guilds in relation to the railway line (Fig 5., MANOVA F_4, 25_ = 1.99; P = 0.09).

## Discussion

Most of the research carried out in the vicinity of roads with heavy traffic has shown that bird densities decrease in such habitats [12,32,40,41]. A similar study carried out along railway tracks performed in nearby meadows corroborates the results obtained on tarred roads [8]. The results of our observations indicate that the railway line does not adversely affect woodland birds during their autumn migration. The results of other experiments carried out in the vicinity of heavily transited railway lines indicated that more birds were found on transects near railway tracks than on those situated further away [30]. Similar results were obtained during a study carried out in eastern Poland in the breeding season: the species richness was greater near a railway line running through a large woodland complex [12]. In addition, a higher density of insectivorous species was found in the vicinity of the tracks. These results were due to the so-called “edge effect” [15]. Habitats on the boundary between a forest and a transport corridor boast a richer flora and fauna [42]; hence, a forest margin with a rich vegetation structure together with railway infrastructure may offer favourable conditions for birds, in contrast to other groups of vertebrates [20]. The electric catenary structures provide good lookout points and resting places for different groups of birds, and the open area of the railway line offers excellent foraging conditions [16]. During the autumn migration, we found no bird species that displayed a particular preference for the neighbourhood of the railway line along a distance of 3.5 km, just as was the case during the breeding season [12]. During the growing and breeding seasons, some bird species find propitious conditions for nesting and breeding along the railway line. During the autumn migration, however, nesting sites are less important for birds than resting points and foraging areas. Therefore, as during the breeding season, we came across more birds from different species in the autumn, regardless of their nesting preferences.

All studies of the impact of noise on birds along roads or railway lines have shown that it plays a key role in their distribution there [5,12,32,40,41]. McClure’s team [4] demonstrated that noise is a key factor affecting the distribution of forest birds along transport corridors. Our research, however, showed that the habitat conditions were the most important. The edge effect and structure of the study area had a major impact on the distribution of birds relative to the railway line, as they did during the breeding period [12]. We showed that, of the various environmental factors taken into consideration, only the canopy cover had an impact on the number of birds and species. A study carried out in spring and summer showed that herb cover was essential to the distribution of birds in the study area [12]. Birds may well find more food in the treetops during the autumn: food resources on the ground are depleted, whereas many insect larvae are available high up in the trees. Feeding in tree crowns is also safer than searching for food on the ground: the probability of a predator attacking on the ground is greater than on a tree. But despite this danger, many bird species continue foraging on the ground [32,42,43]. The results of our research indicate that during consecutive surveys, the numbers of insectivorous birds probably decreased while the numbers of granivorous species increased. Our results resemble those of studies on the impact of road noise on birds during autumn migration [5], but differ from the results from the breeding period when granivorous species were dominant [32]. In both cases, research carried out in the autumn near a tarred road or railway tracks showed insectivorous species to be dominant, through gradually decreasing in favour of granivores [12], because of the favourable habitat and temperatures prevailing near the edge of the forest and the transport corridor [15]. The species richness and forest structure create favourable conditions for numerous species of invertebrates on the edge of woodland [44], a situation that is favourable for insectivores and predators which hunt on the open ground along railway lines [16]. In central Europe, the number of migratory species generally decreases over time, while sedentary species become dominant, which is confirmed by our results.

In conclusion, we did not observe some negative effect of railway traffic on the distribution of woodland birds during the autumn migration period: on the contrary, the effect was rather positive. However, it should be remembered that the results obtained may be a consequence of the influence of two factors: habitat composition and railway operation, therefore in the future it is necessary to clarify the impact of rail traffic and habitats on the presence of birds. The considerable numbers of birds at the edge of the forest near the railway tracks indicates that noise is not a limiting factor for them [12,30]. It is possible that the relatively long time gaps between passing trains do not hinder communication between birds; they become accustomed to the brief disturbances associated with rapidly passing trains, which represent a short-lived, point source of noise [12]. In contrast, the noise produced by road traffic is linear and continuous, and birds tend to avoid such places. Noise produced by trains therefore has a different effect from that coming from highways or other busy roads.

The results presented here may be helpful in the planning and management of railway verges for bird conservation. Our research has shown that the erection of expensive noise barriers is unnecessary. However, the ultimate impact of the railway line will depend on the intensity of traffic. Should there be a significant increase in the number of trains travelling along the railway line under investigation, some noise-reducing measures may be necessary. However, the answer to this question requires further research on railway lines with a much higher traffic volume. In our study area, where we found a greater abundance and diversity of bird species in the forest near the railway tracks, measures must be taken to minimize the possibility of collisions between birds and trains.

## Supporting information

S1 Table Vegetation at the point-count locations in relation to distance from the railway line (points A—30 m, points B—280 m, points C—530 m). Data are presented as median values. The differences between the points were tested using the Kruskal-Wallis test.
(DOCX)

S1 Fig Relationship between A-weighted railway traffic noise (dB) at the point-count locations and distance from the railway in September, October and November. (TIF)

S2 Fig Foraging (Insectivorous; Granivorous-insectivorous) guilds in relation to the successive study months (September, October and November). (TIF)

S3 Fig Migratory and resident guilds in relation to the study months (September, October and November). (TIF)
