# Supplementary material for: Do railway lines affect the distribution of woodland birds during autumn?
Source: PLoS One. 2020 Apr 15;15(4):e0231301. doi: 10.1371/journal.pone.0231301 (PMC7159195; doi:10.1371/journal.pone.0231301)
Supplement: S1 Table — Data are presented as median values. The differences between the points were tested using the Kruskal-Wallis test. (DOCX) [file pone.0231301.s001.docx]

Table 2.Vegetation at the point-count locations in relation to distance from the railway (points A – 30 m, points B – 280 m, points C – 530 m). Data are presented as median values. The differences between points were tested using the Kruskal-Wallis test.

| Variable | points A | points B | points C | *H* _2, 45_ | *P* |
| --- | --- | --- | --- | --- | --- |
| Canopy cover | 5 | 7 | 7 | 12.550 | < 0.05 |
| Tree height (m) | 13 | 14.5 | 13.9 | 3.133 | ns |
| Number of deciduous trees | 2 | 0 | 0 | 10.189 | < 0.05 |
| Number of dead trees | 4 | 3 | 2 | 1.875 | ns |
| Shrub cover | 3 | 3 | 3 | 2.726 | ns |
| Density of shrubs | 115 | 40 | 120 | 2.726 | ns |
| Herb cover | 3 | 1 | 1 | 7.130 | < 0.05 |
| Herb height (cm) | 32.6 | 23.2 | 20 | 4.879 | ns |
