# Supplementary figures and images for: Do railway lines affect the distribution of woodland birds during autumn?
Source: PLoS One. 2020 Apr 15;15(4):e0231301. doi: 10.1371/journal.pone.0231301 (PMC7159195; doi:10.1371/journal.pone.0231301)

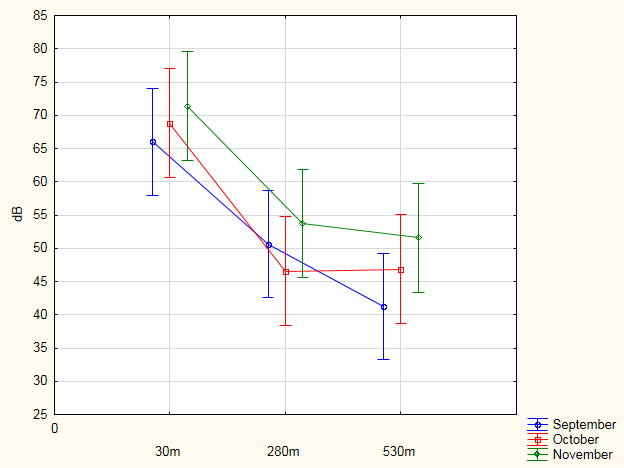

Supplement: S1 Fig — (TIF) [file pone.0231301.s002.tif]

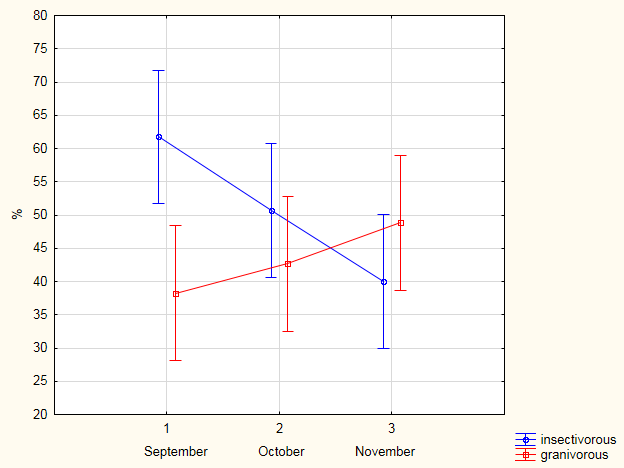

Supplement: S2 Fig — (TIF) [file pone.0231301.s003.tif]

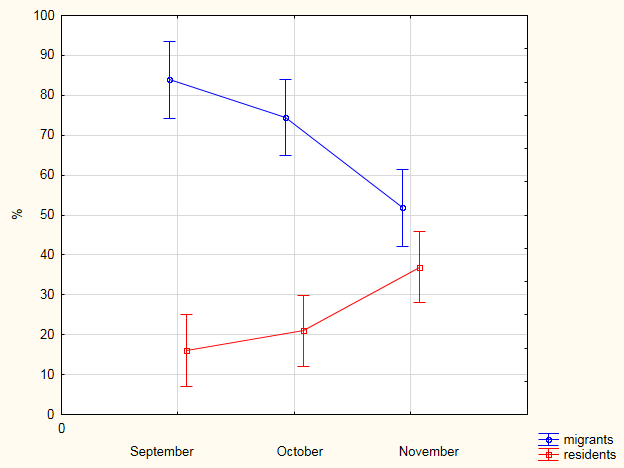

Supplement: S3 Fig — (TIF) [file pone.0231301.s004.tif]
